# Lkb1 loss in regulatory T cells leads to dysregulation of hematopoietic stem cell expansion and differentiation in bone marrow

**DOI:** 10.1002/2211-5463.13536

**Published:** 2023-01-04

**Authors:** Jiadi Chen, Jingru Liu, Huifang Huang

**Affiliations:** ^1^ Clinical Laboratory Fujian Medical University Union Hospital Fuzhou China; ^2^ Central Laboratory Fujian Medical University Union Hospital Fuzhou China

**Keywords:** differentiation, hematopoietic stem cells, homeostasis, Lkb1, Treg

## Abstract

The tumor suppressor Lkb1 is known to regulate the expression of forkhead box P3 (Foxp3), thereby maintaining the levels of Foxp3^+^ regulatory T cells (Treg) that play a crucial role in self‐tolerance. However, the effect of Lkb1 in Treg on hematopoietic stem cells (HSCs) in the bone marrow (BM) remains obscure. Here, we demonstrated that conditional deletion of Lkb1 in Treg causes loss of Treg in the BM, which leads to failure of HSC homeostasis and the abnormal expansion. Moreover, the loss of BM Treg results in dysregulation of other developing progenitors/stem cell populations, leading to the defective differentiation of T cells and B cells. In addition, HSC from the BM with Treg loss exhibited poor engraftment efficiency, indicating that loss of Treg leads to irreversible impairment of HSC. Collectively, these results demonstrated the essential role of Lkb1 in Treg for maintaining HSC homeostasis and differentiation in mice. These findings provide insight into the mechanisms of HSC regulation and guidance for a strategy to improve the outcomes and reduce complications of HSC transplantation.

## Graphical abstract

We demonstrated that conditional deletion of liver kinase b1 (Lkb1) in regulatory T cells (Treg) causes loss of Treg in mouse bone marrow, which leads to failure of hematopoietic stem cell homeostasis and abnormal expansion.

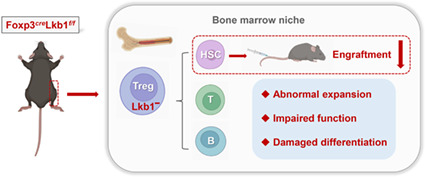

**Abbreviations**
aGVHD: acute graft‐versus‐host disease
BM: bone marrow
BMM: bone marrow microenvironment
CMP: common myeloid progenitor
GMP: granulocyte‐macrophage progenitor
HPC: hematopoietic progenitor cells
HSC: hematopoietic stem cells
KO: knock‐out
Lkb1: liver kinase b1
LT‐HSC: long‐time HSC
MEP: megakaryocyte erythroid progenitor
ST‐HSC: short‐time HSC
TGF‐β: transforming growth factor‐β
Treg: regulatory T cells
WT: wild‐type

The HSC niche, namely the bone marrow microenvironment (BMM), is composed of various cell types, including Foxp3^+^ regulatory T cells (Treg) [1, 2], indicating that Treg plays a crucially important role in maintaining HSC homeostasis. Several groups demonstrated that the CD150^+^ Treg subpopulation localizes in the hematopoietic stem cell (HSC) niche and maintains HSC quiescence and immune privilege via CD73 [3]. Furthermore, loss of Treg in the bone marrow (BM) leads to failure of B‐cell lymphopoiesis and Treg inhibits CD34^+^ cells differentiation into NK cells, indicating that Treg affects the differentiation of HSC [4].

In addition, accumulating evidence suggests that Treg can prevent the complications of allogeneic transplantation, especially acute graft‐versus‐host disease (aGVHD), a significant adverse complication following allogeneic hematopoietic cell transplantation [5, 6, 7, 8]. Adaptive transfer of donor Treg could abrogate aGVHD without reducing graft‐versus‐tumor effects in mouse models and clinical treatment [9, 10]. Therefore, Treg emerged as an essential factor for HSC homeostasis and reconstitution.

Liver kinase b1 (*Lkb1*) is a tumor suppressor that has been reported to be associated with many different types of cancer [11, 12, 13]. Moreover, *Lkb1* also plays a crucial role in immune cells, including dendritic cells [14, 15, 16, 17], macrophages, [18], and Treg [19, 20]. We previously demonstrated that the Lkb1 signaling pathway is essential for maintaining Treg homeostasis by stabilizing *Foxp3* expression [21]. *Foxp3* is specifically expressed in Treg and is required for their differentiation from naive CD4^+^T cells [22]. Conditional deletion of *Lkb1* in the Treg of mice resulted in a dramatic decrease of Treg with consequent development of autoimmune disease due to the STAT4‐mediated methylation of conserved noncoding sequence 2 in the *Foxp3* locus. Thus, Lkb1 is necessary for maintaining Treg cell lineage identity.

Therefore, in the present study, we used the same mouse model with conditional deletion of *Lkb1* in Treg to determine the effect on HSC expansion and differentiation into T cells and B cells. Furthermore, we verified lymphopoiesis of T‐ and B‐lineage cells by assessing the engraftment efficiency of HSC derived from BM with Treg loss. These results can provide another insight into the relevance of Treg for maintaining the HSC niche with potential clinical application to protect against aGVHD as a significant complication of allogeneic hematopoietic cell transplantation.

## Materials and methods

### Mice

**Fig. 1:**
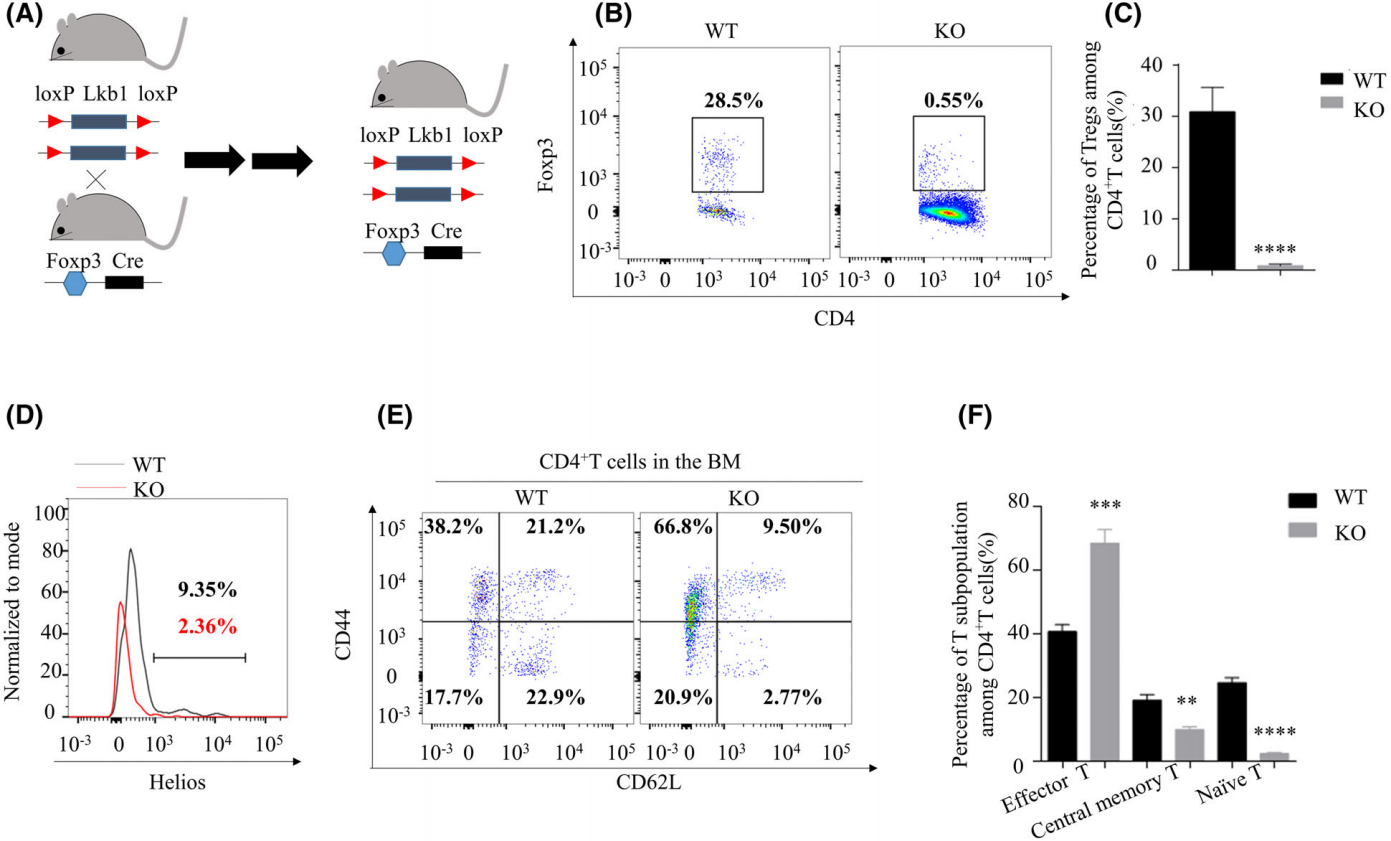
*Lkb1* conditional deletion in Treg induces the loss of Treg in the bone marrow (BM) and alters the CD4^+^T subpopulations. (A) The generation of *Lkb1* conditional deletion in Treg mice. (B) Flow cytometric analysis of Treg cell ratios among CD4^+^T cells in the BM from wild‐type (WT) and knock‐out (KO) mice. (C) Statistical comparison of the proportions of Treg among CD4^+^T cells between WT and KO BM (*n* = 3). (D) Helios expression in the BM Treg from WT and KO mice. (E) The flow cytometric analysis of CD62L^low^ CD44^high^ effector cells, CD62L^+^CD44^+^ central memory cells and CD62^+^CD44^−^ naïve cells in the BM among CD4^+^T cells from WT and KO mice. (F) Statistical comparison of the proportions of effector cells, central memory cells, and naïve cells among CD4^+^T cells in the BM from WT and KO mice (*n* = 3). The results were presented as the mean ± SEM, *P < 0.05, **P < 0.01, ***P < 0.001, ****P < 0.0001, by Student's *t*‐test (C, F). All results were performed with at least three independent experiments.

To investigate the role of Treg in regulating hematopoiesis in the BM, we generated *Foxp3* ^*Cre*^ *Lkb1* ^*f/f*^ knock‐out (KO) mice, with *Lkb1*conditionally deleted in Treg, as previously described (Fig. 1A) [21]. In brief, *Lkb1* ^*f/f*^ mice were crossed with *Foxp3* ^*Cre*^ mice (both purchased from Jackson Laboratories, Bar Harbor, ME, USA) to generate *Foxp3* ^*Cre*^ *Lkb1* ^*f/f*^ KO mice. Wild‐type (WT) *Lkb1* ^*f/f*^ mice matched with the KO mice in age and sex were selected as controls. All mice were used in experiments at about 4 weeks old. All mice were maintained under specific pathogen‐free barrier facilities. The animal use protocol listed below has been reviewed and approved by the Institutional Animal Care and User Committee and Animal Ethical and Welfare Committee (No. SYXK Jin 2015‐0001) at the Institute of Hematology, Chinese Academy of Medical Sciences.

### Transplantation experiments

To evaluate whether the loss of Treg in the BM leads to HSC defects *in vivo*, we performed competitive BM transplantation assays since BM transplantation provides a powerful tool to evaluate the capacities of HSC self‐renewal and differentiation. BM cells derived from KO mice (CD45.2 genetic background) and WT mice (CD45.1/CD45.2 genetic background) were sorted by Lin^−^ CD3^−^ CD19^−^ markers, respectively, and mixed at a 1 : 1 ratio. The mixed cells were then injected into male B6 mice (CD45.1 genetic background), which were irradiated with 800 cGy split into two doses on day 0. At 4 weeks after transplantation, flow cytometric analysis was performed to determine the efficiency of engraftment.

### Antibodies and reagents

The surface markers included FITC‐CD3 (MA1‐7640, eBioscience, San Diego, CA, USA), APC‐Cy7‐CD4 (100413, Biolegend, San Diego, CA, USA), APC‐CD4 (100411, Biolegend), PerCP‐CD8 (100,731, Biolegend), PE‐Cy7‐CD8 (100721, Biolegend), PE‐Foxp3 (12‐5773‐82, eBioscience), APC‐Helios (17‐9883‐42, eBioscience), PerCP‐CD44 (103035, Biolegend), FITC‐CD62L (104405, Biolegend), FITC‐lin (22‐7770‐72, eBioscience), PerCP‐CD34 (50‐0341‐82, eBioscience), APC‐Sca‐1 (17‐5981‐82, eBioscience), PE‐Sca‐1 (12‐5981‐82, eBioscience), APC‐c‐kit (17‐1171‐82, eBioscience), PE‐c‐kit (12‐1171‐82, eBioscience), PE‐B220 (12‐0452‐82, eBioscience), FITC‐IgM (11‐5790‐81, eBioscience), PE‐Cy7‐CD19 (12‐0193‐82, eBioscience), APC‐CD19 (17‐0193‐82, eBioscience), PE‐Cy7‐CD16/32 (25‐0161‐82, eBioscience), PE‐Cy7‐CD45 (25‐0451‐82, eBioscience), PerCP‐5.5‐CD45.2 (45‐0454‐82, eBioscience), and APC‐Cy7‐CD45.1 (17‐0453‐82, eBioscience) obtained from Invitrogen. Intracellular staining with PE‐Foxp3 (72‐5775‐40, eBioscience) was performed with Foxp3 staining kits (Invitrogen, Carlsbad, CA, USA).

### Flow cytometry

The BM was harvested by flushing the long leg bones with phosphate‐buffered saline containing 2% fetal bovine serum. The prepared samples were then stained according to standard procedures for flow cytometry. Data were acquired on an LSR Fortessa (BD Biosciences, Franklin Lakes, NJ, USA) or FACS Canto II (BD Biosciences) system and analyzed with flow jo 10.8.1 software (Tree Star, Franklin Lakes, NJ, USA).

### Statistical analysis

An unpaired, two‐tailed, Student's *t*‐test was applied for comparisons between the KO and WT groups using graphpad prism 9.0 software. *P* values < 0.05 were considered statistically significant (*P < 0.05; **P < 0.01; ***P < 0.001; ****P < 0.0001); NS, not significant.

## Results

### *Lkb1* conditional deletion in Treg induces the loss of Treg in the bone marrow and alters the CD4 ^+^T subpopulations

*Lkb1* conditional deletion in Treg caused unstable *Foxp3* expression and induced the loss of Treg concerning percentages among CD4^+^T cells (Fig. 1A–C). In addition, the Treg in the BM from KO mice showed lower expression levels of Helios, a characteristic marker of naïve Treg cells [23] (Fig. 1D). Analysis of the CD4^+^T subpopulation in the BM showed significantly increased frequencies of CD62L^low^CD44^high^ T cells in the BM from KO mice, indicating that CD4^+^T cells in the BM acquired an activated phenotype after the loss of Treg. Furthermore, the frequencies of the CD62L^+^CD44^+^ central memory T cells [24] and CD62L^+^CD44^−^ naïve T cells among the CD4^+^T population were decreased in BM of KO compared with those from the WT mice (Fig. 1E,F).

### Lkb1 loss in Treg in the bone marrow leads to abnormality of bone marrow development

**Fig. 2:**
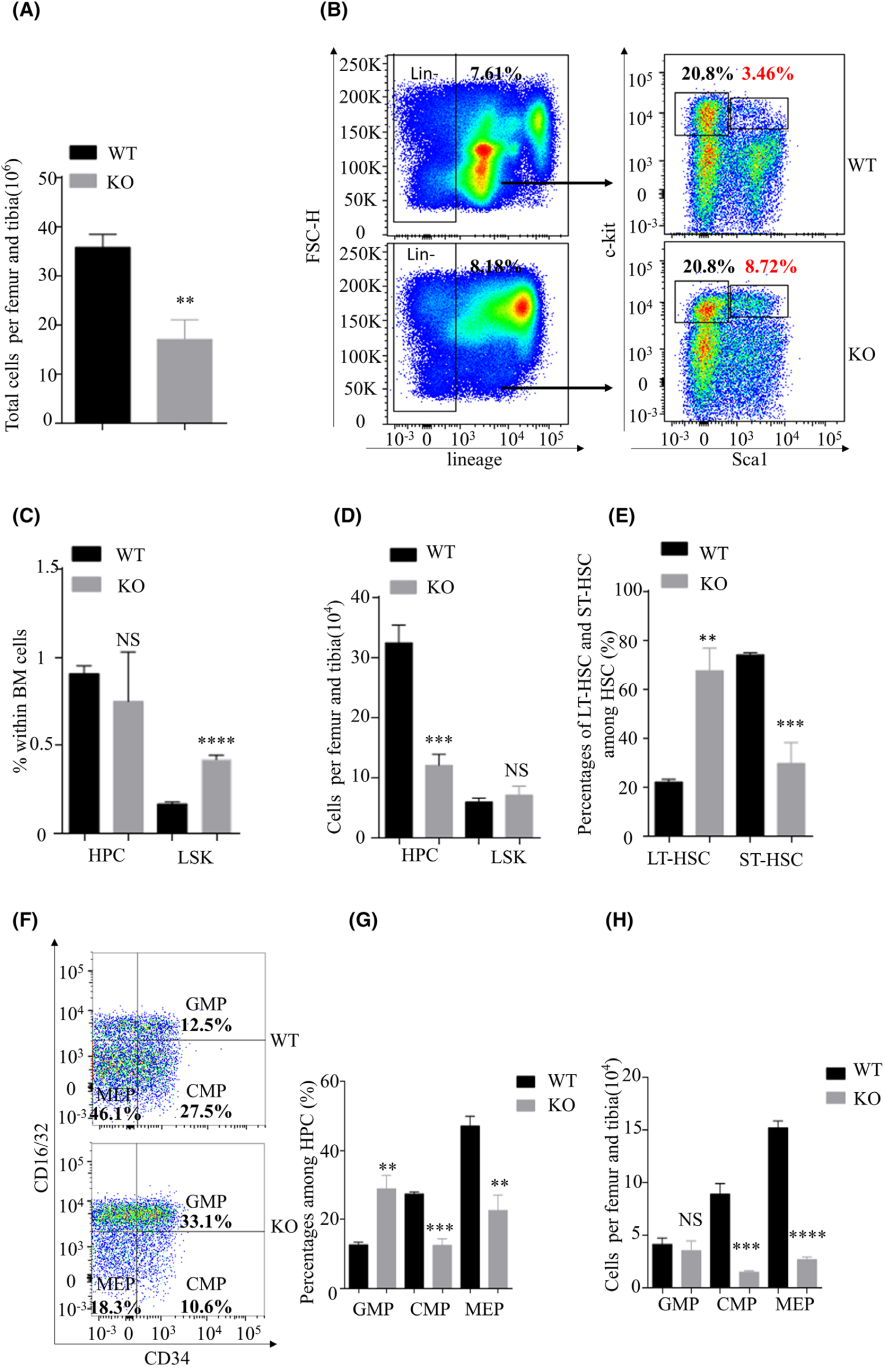
Lkb1 loss in Treg in the bone marrow (BM) leads to abnormality of BM development. (A) Statistical comparison of the absolute numbers of total BM cells between wild‐type (WT) and knock‐out (KO) BM (*n* = 3). (B) Flow cytometric analysis of hematopoietic progenitor cell (HPC) and hematopoietic stem cell (HSC) in the BM cells from WT and KO mice. (C) Statistical comparison of the proportions of HPC and HSC among BM total cells between WT and KO BM (*n* = 3). (D) Statistical comparison of the absolute numbers of HPC and HSC between WT and KO BM (*n* = 3). (E) Statistical comparison of the proportions of long‐time HSC and short‐time HSC among HSC between WT and KO BM (*n* = 3). (F) Flow cytometric analysis of HPC subpopulation in the BM from WT and KO mice. (G) Statistical comparison of the proportions of GMP, CMP, and MEP among HPC between WT and KO BM (*n* = 3). (H) Statistical comparison of the numbers of GMP, CMP, and MEP per femur and tibia between WT and KO BM (*n* = 3). The results were presented as the mean ± SEM, *P < 0.05, **P < 0.01, ***P < 0.001, ****P < 0.0001, by Student's *t*‐test (A, C, D, E, G, H). All results were performed with at least three independent experiments.

The number of total BM cells derived from KO mice was dramatically reduced compared with WT mice (Fig. 2A), indicating a defect in BM development. A significantly increased percentage of HSC among BM was detected in the KO mice. In contrast, there is no noticeable difference in the percentage of hematopoietic progenitor cells (HPCs) among BM total cells between KO and WT mice (Fig. 2B,C). However, the absolute number of HSC and HPC did not alter and significantly decreased. This is due to the sharply reduced number of total BM cells (Fig. 2D). These results indicated that the BM of mice with the loss of Treg had impaired hematopoiesis, which may influence the resident HSC and HPC populations.

To further define the characteristics of HSCs, we also determined the frequencies of long‐time HSC (LT‐HSC) and short‐time HSC (ST‐HSC) and found the percentages of LT‐HSC were significantly increased. In contrast, the percentages of ST‐HSC were reduced in the BM of KO mice compared with those of their WT littermates (Fig. 2E). HPC can be divided into common myeloid progenitor (CMP), megakaryocyte erythroid progenitor (MEP), and granulocyte macrophage progenitor (GMP). The BM with *Lkb1* conditionally deleted in Treg showed significantly decreased percentages and absolute numbers of CMP and MEP compared with those of their WT littermates. By contrast, the BM from KO mice displayed a dramatic increase in the percentage of GMP compared with those of the WT BM, whereas there was no difference in the absolute numbers of GMP between WT and KO mice (Fig. 2F–H). These data suggested that Treg is essential in maintaining HSC and multiple progenitor/stem cells.

### Lkb1 loss in Treg resulted in the abnormal differentiation of T‐ and B‐ cell lineages in the bone marrow

**Fig. 3:**
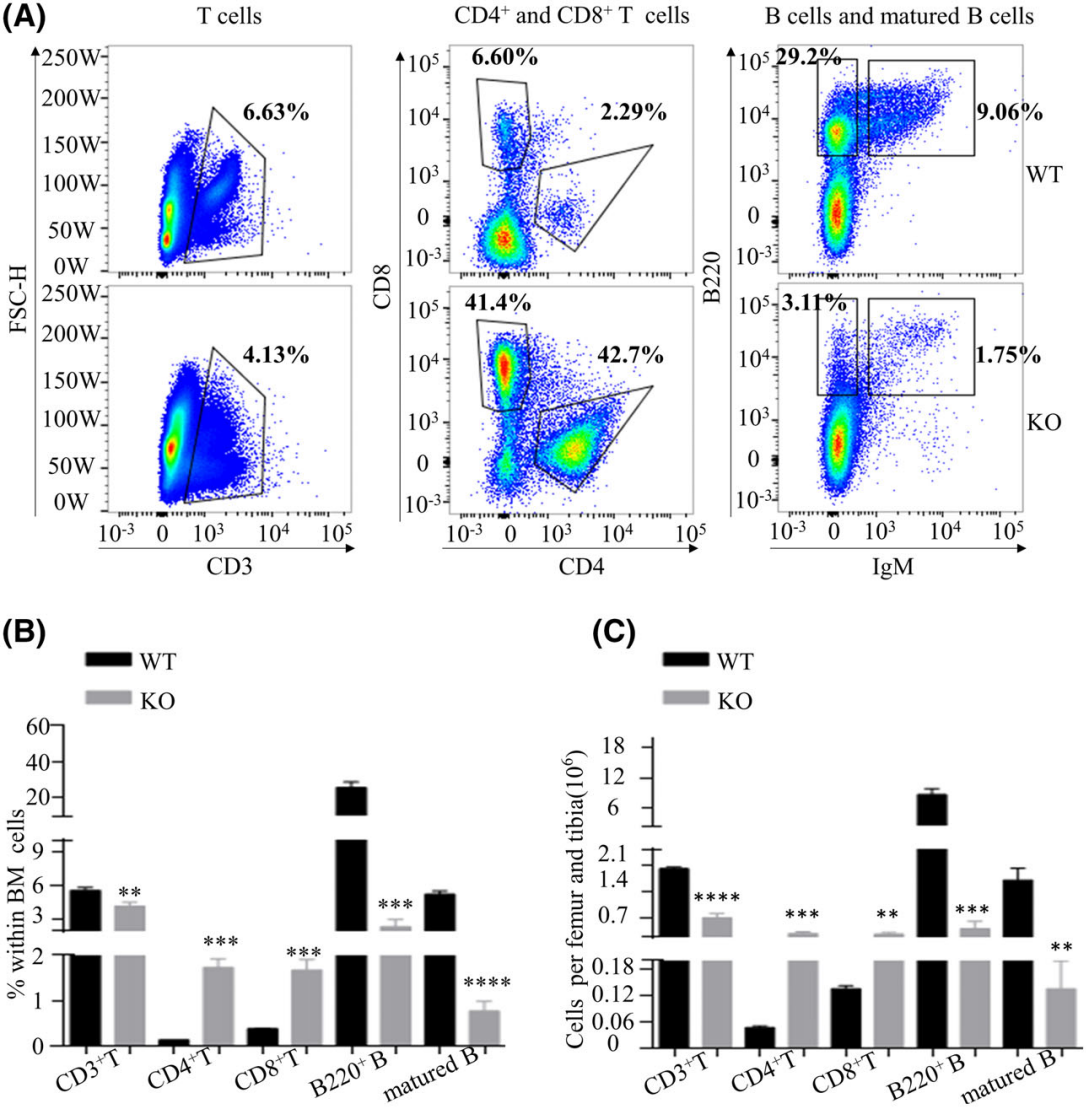
Lkb1 loss in Treg resulted in the abnormal differentiation of T‐ and B‐cell lineages in the bone marrow (BM). (A) The gating strategy and representative FACS data of total BM cells from wild‐type (WT) mice and knock‐out (KO) mice including T cells, CD4^+^T cells, CD8^+^T cells, and B cells. (B) Statistical comparison of the proportions of multiple cells among the total BM cells between WT and KO BM (*n* = 3). (C) Statistical comparison of the absolute numbers of multiple cells in the BM between WT and KO BM (*n* = 3). The results are presented as the mean ± SEM, *P < 0.05, **P < 0.01, ***P < 0.001, ****P < 0.0001, NS meaning not significant by Student's *t*‐test (B, C). All results were performed with at least three independent experiments.

T cells and B cells were determined to further investigate the differentiation of HSCs. The BM from KO mice showed a slight but significant decrease in the percentages of CD3^+^T cells. In contrast, the percentages and numbers of CD4^+^T and CD8^+^T cells dramatically increased, indicating that a decrease in Treg influenced HSC differentiation into T cells. We also observed a defect of B cell differentiation and lymphopoiesis in BM derived from KO mice (Fig. 3A). Both the percentages and absolute numbers of B220^+^cells and mature B cells were sharply decreased in the BM derived from KO. Myeloid cells (Gr1^+^CD11b^+^) and macrophages which are critical immune cells in the BM were also dysregulated in the BM derived from KO mice (Fig. 3B,C). Taken together, our results indicated that Treg maintains the HSC pool and their normal differentiation into T cells and B cells.

### Lkb1 loss in Treg in the bone marrow showed poor engrafting efficiency

**Fig. 4:**
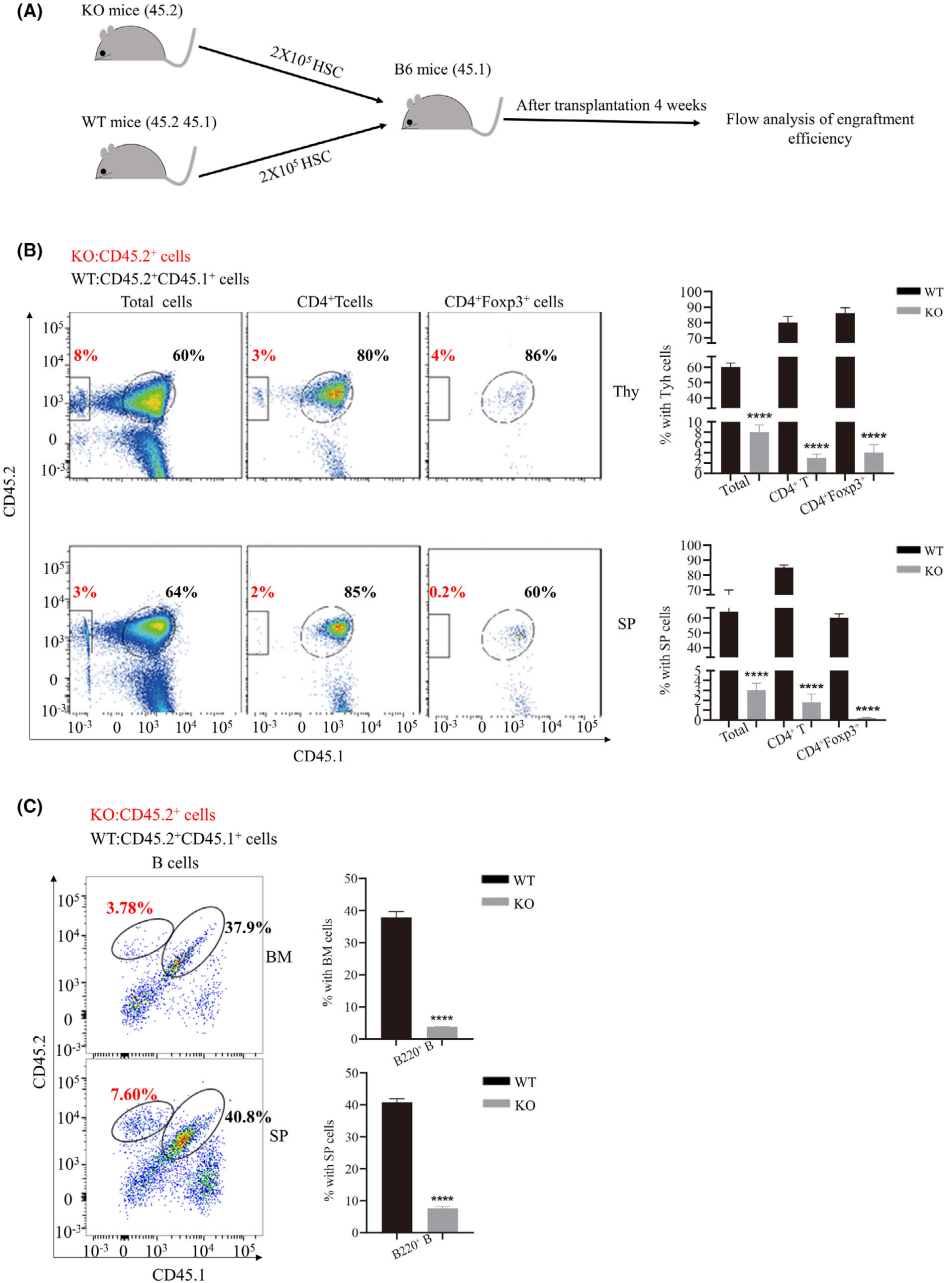
Lkb1 loss in Treg in the bone marrow (BM) showed poor engrafting efficiency. (A) Experimental scheme of competitive transplantation assay. The same number (10^5^ cells) of Lin^−^CD3^−^CD19^−^BM cells from knock‐out (KO) mice (CD45.2) and wild‐type (WT) mice (CD45.1/CD45.2) were co‐injected into lethally irradiated B6 mice (45.1). Then the flow analysis was performed after transplantation after 4 weeks. (B) The flow cytometric analysis of CD45.2 cells and CD45.1/CD45.2 cells in the total cells, CD4^+^T cells, and CD4^+^Foxp3^+^Tregs in the spleen and thymus. And statistical comparison of the proportions of multiple cells among the total cells in the spleen and thymus between WT and KO (*n* = 5). Note that the CD45.2 cells derived from KO mice demonstrated poor engraftment efficiency and failure to BM reconstitution compared with CD45.1/CD45.2 cells derived from WT mice. (C) The flow cytometric analysis of CD45.2 cells and CD45.1/CD45.2 cells in the CD19^+^B cells in the spleen and BM and a statistical comparison of the proportions of multiple cells among the total cells in the BM and spleen between WT and KO (*n* = 5). Note that CD45.2 cells derived from KO mice demonstrated poor engraftment efficiency and defect of B cells differentiation compared with CD45.1/CD45.2 cells derived from WT mice. The results were presented as the mean ± SEM, *P < 0.05, **P < 0.01, ***P < 0.001, ****P < 0.0001, by Student's *t*‐test. All results were performed with at least three independent experiments.

Lin^−^CD3^−^CD19^−^ were sorted from KO (CD45.2 genetic background) and WT (CD45.1/CD45.2 genetic background) mice, respectively. Then mixed with a ratio of 1 : 1 and transplanted into host mice (CD45.1 genetic background). About 4 weeks after transplantation, flow cytometric analysis was performed (Fig. 4A). The total spleen and thymus cells showed much lower percentages of CD45.2 cells derived from the KO mice than those of CD45.1/CD45.2 cells derived from the WT mice. CD4^+^T cells, as well as CD4^+^Tregs derived from KO mice, were much lower than that derived from WT mice (Fig.4B). Moreover, the percentages of B cells derived from KO mice were much lower than that of WT mice in the BM and spleen (Fig. 4C). In fact, the BM derived from KO mice failed to reconstitute in the recipient mice. These data suggested that the loss of Treg leads to an irreversible defect of HSC and a normal BM microenvironment cannot rescue HSC defect.

## Discussion

*Lkb1* has been shown to maintain the homeostasis of *Foxp3* ^+^ Tregs in mice, which are essential for promoting self‐tolerance and suppressing autoimmunity [4, 20]. Here, we further demonstrated that *Lkb1* in Treg is also required to maintain HSC homeostasis and is necessary for the differentiation of BM cells, including T cells and B cells. In addition, HSC from mice with *Lkb1* conditional deletion in Treg displayed poor engraftment efficiency, which indicated that Treg leads to irreversible impairment for HSC.

Treg is widely recognized for its essential role in maintaining HSC quiescence. Indeed, conditional deletion of Treg in the BM led to the abnormal expansion of HSCs. CD150^high^Tregs resident in the HSC niche could maintain HSC quiescence by protecting the cells from oxidative stress via cell–cell contact, while CD150^low^Tregs have little impact on HSC numbers [3]. Moreover, Treg can protect against the development of aGVHD and modify the host BM environment to facilitate donor HSC engraftment, further indicating their role in regulating HSC homeostasis [25, 26, 27, 28]. In the present study, we confirmed that the loss of Treg in the BM results in an abnormal and dysfunctional HSC population.

The loss of Treg in the BM leads to dysfunctional BMM, including altered production of cytokines, which are critical for differentiating cells to multiple lineages. For example, transforming growth factor (TGF)‐β can induce the differentiation of HSCs towards the myeloid lineage rather than the lymphoid lineage [29], and Treg could inhibit HSC differentiation into natural killer cells by producing TGF‐β [30]. IL‐7 and CXCL12 are critical cytokines for B‐cell differentiation [31], and the loss of Treg induces a reduction of IL‐7 production by the ICAM1^+^ stromal [4]. Indeed, we observed a defect of B‐cell differentiation in the BM with the loss of Treg. CCL‐6 has previously been reported to regulate the differentiation of myeloid cells, macrophages, B cells, and CD4^+^ lymphocytes [32]. We also observed dysregulated differentiation in T cells, myeloid cells, and macrophages with loss of Treg, which may result from the dysregulation in the production of specific cytokines and require further studies for confirmation. Thus, we suspect that the BMM to promote the differentiation of resident cells became more susceptible to immune cell activation after the loss of Treg.

Importantly, we found that the BM from KO mice exhibited poor engraftment efficiency and failed to establish BM chimera in the recipient mice. We found a defect of T‐lineage and B‐lineage lymphopoiesis derived from the KO BM in the recipient mice, which indicates that the loss of Treg not only impacts the population of HSCs but also regulates HSC homing and/or engraftment. Further investigations to uncover these mechanisms are warranted.

Taken together, we demonstrate that *Lkb1* in Treg maintains HSC/HPC quiescence, function, and differentiation. Our data provide critical insights into the biology and function of Treg in the BM, while highlighting potential clinical applications for treating or preventing aGVHD after transplantation.

## Conclusions

Taken together, we demonstrate that *Lkb1* in Treg maintains HSC/HPC quiescence, function, and differentiation. Our data provide critical insights into the biology and function of Treg in the BM, while highlighting potential clinical applications for the treatment or prevention of aGVHD after transplantation.

## Conflict of interest

The authors declare no conflict of interest.

## Author contributions

JC, JL, and HH designed the research and analyzed the data; JC, JL, and HH wrote the manuscript. JC and JL performed the experiments. All authors provided approval of the final version.

## Data Availability

The additional data used to arrive at these conclusions can be obtained from the corresponding author on reasonable request.
